# Chemical Structure and Side Reactions in Polyurea Synthesized via the Water–Diisocyanate Synthesis Pathway

**DOI:** 10.3390/polym15173524

**Published:** 2023-08-24

**Authors:** Theodor Stern

**Affiliations:** Department of Chemical Engineering, Biotechnology and Materials, Faculty of Engineering, Ariel University, Ariel 40700, Israel; theodorst@ariel.ac.il

**Keywords:** polyurea, crosslinking, expanded polyurea, FTIR, NMR, polymerization mechanism

## Abstract

Industrial polyureas are typically synthesized using diisocyanates via two possible alternative pathways: the extremely quick and highly exothermal diamine–diisocyanate pathway and the relatively slow and mild water–diisocyanate pathway. Although polyurea synthesis via the water–diisocyanate pathway is known and has been industrially applied for many decades, there is surprisingly very little analytical information in the literature in relation to the type and extent of the occurring side reactions and the resulting chemical structures following this synthesis pathway. The synthesis of polyureas exhibiting very high concentrations of carbonyl-containing groups resulted in strong and accurate diagnostic analytical signals of combined FTIR and solid-state ^13^C NMR analysis. Despite the strictly linear theoretical chemical structure designed, the syntheses resulted in highly nonlinear and crosslinked polymers. It was analytically found that the water–diisocyanate pathway preferentially produced highly dominant and almost equal contents of both biuret structures and tertiary oligo-uret structures, with a very small occurrence of urea groups. This is in strong contrast with the chemical structures previously obtained via the diamine–diisocyanate polyurea synthesis pathway, which almost exclusively resulted in biuret structures. The much slower reaction and crosslinking rate of the water–diisocyanate synthesis pathway enabled the further access of isocyanate groups to the already-formed secondary nitrogens, thus facilitating the formation of complex hierarchical tertiary oligo-uret structures.

## 1. Introduction

Polyureas inherently contain at least one urea group in the polymer repeating unit, the presence of which is known to result in polymers of enhanced mechanical properties and are industrially synthesized and processed for a vast and highly versatile range of applications. 

Industrial polyureas are typically synthesized using diisocyanates via two possible alternative pathways: the extremely quick and highly exothermal diamine–diisocyanate pathway and the relatively slow and mild water–diisocyanate pathway [1]. 

The diamine–diisocyanate synthesis pathway, according to which the urea groups in polyureas are obtained through the reaction between a diisocyanate and a primary diamine, is very commonly used in industry, given the extremely fast room-temperature reaction kinetics of this synthesis pathway. Due to the high nucleophilicity of the primary amines, the reactivity of isocyanates with primary amines to form polyureas is orders of magnitude faster than the reactivity of the isocyanates with primary alcohols to form polyurethanes [2,3]. Consequently, the diamine–diisocyanate polyurea synthesis readily occurs at room temperature and does not require the use of a catalyst [4,5]. The very fast kinetics of these reactions enable the development of industrial reaction injection molding (RIM) processing, which is of particularly high significance in the automotive industry [5,6]. Also well-known are the reactive spray-coating applications, which exhibit enhanced properties such as high ballistic impact resistance [6,7,8,9,10]. The synthesis of polyurea block copolymers via the diamine-mediated pathway, containing various types of soft segments, led to relatively flexible polyureas being obtained for various applications such as biomedical applications [11,12,13,14,15,16,17]. 

The water–diisocyanate synthesis pathway occurs through the reaction of the isocyanate groups with water to result in a nonstable intermediate carbamic acid, which converts to a primary amine group by losing a CO_2_ molecule. The resulting primary amine in turn reacts with an additional isocyanate group, resulting in a urea linkage [1]. The water–diisocyanate polyurea synthesis pathway occurs under much milder conditions and very significantly slower reaction kinetics (as compared with the diamine-mediated synthesis pathway), due to the above-mentioned characteristic intermediate reaction steps of this synthesis pathway [1]. This synthesis pathway is mainly performed in industrial processes for the production of foams through CO_2_ gas-mediated polymer expansion and the synthesis of expanded poly(urethane-urea) copolymers, block copolymers, and poly(ether-urethane-urea) block copolymers [1,18,19,20,21]. 

The inherently very high reactivity of the isocyanate groups used in polyurea synthesis is widely known to result in side reactions occurring between isocyanate groups and the secondary amines of already-formed urea groups [1,2,10,22]. Also, since bifunctional isocyanates (i.e., diisocyanates) interact with multi-urea-containing growing chains (i.e., polyfunctional molecules) during polyurea synthesis, these side reactions predominantly result in the significant crosslinking of the final polymer [1,2,10,22]. The occurrence of side reactions, as well as the degree of crosslinking, are known to highly affect the mechanical properties and many other properties of polyureas and polymers in general [22]. 

Although polyurea synthesis via the water–diisocyanate pathway (involving the reaction of diisocyanates and water) is well known and has been industrially applied for many decades, there is surprisingly very little analytical information in the literature in relation to the type and extent of the occurring side reactions and the resulting chemical structures following this synthesis pathway. 

The present research focused on a direct investigation and diagnostic determination of the type and extent of the side reactions that occur and the resulting chemical structures obtained in polyurea synthesis performed via the water–diisocyanate polyurea synthesis pathway, using a combination of FTIR and solid-state ^13^C NMR analysis. 

The research results revealed the types of side reactions and the resulting chemical structures obtained via the water–diisocyanate polyurea synthesis pathway and were compared with the significantly different results previously obtained via the diamine–diisocyanate polyurea synthesis pathway. The suggested mechanisms and spectral diagnostic parameters were further experimentally confirmed through the synthesis and analysis of a completely new type of polymer, inherently exhibiting oligo-uret structures as part of the polymer repeating unit. 

## 2. Experimental Section

### 2.1. Materials

The materials included hexamethylene diisocyanate (purum, ≥98.0%; Sigma, St. Louis, MO, USA); water—HPLC grade (Merck, Burlington, MA, USA); ethyl stannous hexanoate (Tin (II) 2-ethylhexanoate 92.5–100%) (Sigma); urea (ACS reagent, 99%; Sigma); and KBr powder for FTIR analysis (FTIR grade, ≥99%; Sigma) (Sigma-Aldrich Israel Ltd., an affiliate of Merck KGaA, Darmstadt, Germany—Rehovot, Israel).

### 2.2. Instrumentation

FTIR analyses were carried out on a PerkinElmer Spectrum BX FTIR Spectrometer (instrument resolution: 4 cm^−1^). Small amounts (8–10 mg) of each polymer resulting from the performed syntheses were ground into a fine powder and mixed with 140 mg of dried KBr powder. The mixture was ground again in a clean agate mortar until a homogeneous fine powder was obtained. KBr pellets were prepared. Following a background measurement, FTIR measurements were performed by placing the KBr mixture pellets in the path of the instrument beam. The measurements were performed in the 450–4000 cm^−1^ range. A total of 16 scans were performed in each measurement. All spectra were measured in the absorbance mode. 

The solid-state ^13^C NMR experiments were performed on a Bruker Avance III 400 MHz narrow-bore spectrometer, using a 4 mm BB/^1^H/^19^F magic-angle spinning (SB MAS) probe. ^13^C cross-polarization magic-angle spinning (CPMAS) experiments were carried out at a spinning rate of 8 KHz, using a 2.9 μs ^1^H 90° pulse, 2K data points, and a 2 ms ramped-CP period. Proton decoupling using the small-phase incremental alteration (SPINAL) composite pulse sequence at a field of 86 KHz was used during both the acquisition and the 3 s cycle delay between the acquisitions. 

Samples of about 200 mg of the polymers resulting from the performed syntheses, were ground into a fine powder and passed through a fine sieve, in order to obtain a smaller grain size and smaller grain size distribution, as required for the solid-state ^13^C NMR measurements. 

Optical microscopy studies of the expanded polyurea samples were performed on a Nikon H550S optical microscope (Japan), which is equipped with an INVENIO 5SCIII 5M Pixel USB3 color digital camera, connected to Deltapix software. Small and thin polymer fragments were broken off with a hammer and a small chisel and placed in the microscope viewing field. Only focus and light intensity were adjusted during the microscopy studies. 

### 2.3. Polymer Syntheses

Polyurea synthesis via the water–diisocyanate pathway, was performed by reacting hexamethylene diisocyanate (HDI) with water (H_2_O), at a molar ratio of 1:1, resulting in poly(hexamethylene urea). The reagents and molar ratio were chosen to obtain a very high concentration of carbonyl-containing groups in the polymer, which are separated by only six carbon atoms, so as to result in strong and accurate diagnostic analytical signals of combined FTIR and solid-state ^13^C NMR analysis.

The synthesis was carried out in bulk, in a cylindrical 100 mL glass reactor. Polyurea synthesis was calculated to obtain 25 g of the final polymer. 

The HDI was removed from refrigeration prior to synthesis, for a period long enough to reach room temperature, in order to prevent atmospheric water vapor condensation when opening the container. The glass reactor was dried at 105 °C prior to synthesis, in order to remove any adsorbed atmospheric water. 

The HDI was weighed and directly added to the reactor. Briefly, 0.2 mL of ethyl stannous hexanoate catalyst was added to the HDI-containing reactor. The H_2_O reagent was weighed separately and added to the HDI in the reactor, under continuous magnetic stirring. The reactor was heated to 65 °C, using a laboratory magnetic stirrer heating plate and an oil bath, the temperature of which was adjusted before starting the synthesis, using a thermometer. The release of CO_2_ bubbles in the reactor content occurred for approximately 20 min. 

The bubble release occurred as a consequence of the occurrence of the reaction of the isocyanate groups with water, resulting in nonstable intermediate carbamic acid and, finally, a primary amine group, by losing a CO_2_ molecule. 

When the CO_2_ bubbles’ release rate decreased, the heating process was interrupted, and the synthesis was continued under continuous magnetic stirring. The gradual solidification of the reactor content caused the halting of magnetic stirring. Thus, further stirring of the reactor content was performed manually, using a strong stainless steel spatula (the reactor was maintained in the oil bath and removed toward the end of the manual stirring). Manual stirring was continued until polymer solidification in the reactor reached a very high rigidity, which prevented the possibility of further manual mixing. The reactor was covered during the magnetic-stirring stages and opened during the manual-stirring stage. 

The synthesis of poly(hexamethylene oligo-uret), via diisocyanate and urea: As the urea reagent is a diamine, the synthesis in this study was performed under the exact same conditions as I have recently described for the synthesis of polyurea via the diamine–diisocyanate synthesis pathway [23]. The synthesis was carried out in bulk, in a cylindrical 100 mL glass reactor. Polyurea synthesis was calculated to obtain 25 g of the final polymer.

The synthesis was performed at a diisocyanate–urea molar ratio of 1:1. The HDI was removed from refrigeration prior to synthesis, for a period long enough to reach room temperature, in order to prevent atmospheric water vapor condensation when opening the container. The glass reactor was dried at 105 °C prior to synthesis in order to remove any adsorbed atmospheric water. 

The HDI was weighed and directly added to the reactor. Briefly, 0.2 mL of ethyl stannous hexanoate catalyst was added to the HDI-containing reactor. The urea reagent was weighed separately and slowly added to the HDI in the reactor, for approximately 10–15 s, under continuous magnetic stirring at room temperature. The reactor was open during the synthesis process. The reaction was extremely exothermal and quick. Due to the gradual solidification of the reactor content, magnetic stirring was halted. Thus, further stirring of the reactor content was performed manually, using a strong stainless steel spatula. Manual stirring was continued until polymer solidification in the reactor reached a very high rigidity, which prevented the possibility of further manual mixing. 

The synthesis performed in this research using the diamine (urea) was highly exothermal. Thus, in order to prevent extreme overheating during the reaction, the reactor was intermittently immersed in an ice container for very short periods (2–5 s), during the manual stirring process. Special attention and caution during the intermittent ice immersions were required, since the high thermal shock may cause reactor breaking. 

## 3. Results and Discussion

In order to obtain significantly strong diagnostic analytical measurement signals, the polyurea syntheses in the present research were designed to contain significantly small R groups and, consequently, a significantly high concentration of the newly formed chemical structures and their further side-reacted derivatives. Accordingly, polyurea was hereby synthesized by reacting hexamethylene diisocyanate (HDI) with water (H_2_O) at a 1:1 molar ratio, resulting in poly(hexamethylene urea). The synthesis resulted in the formation of a solid polymer, the weight of which was close to the stoichiometrically calculated weight. 

**Scheme 1:**
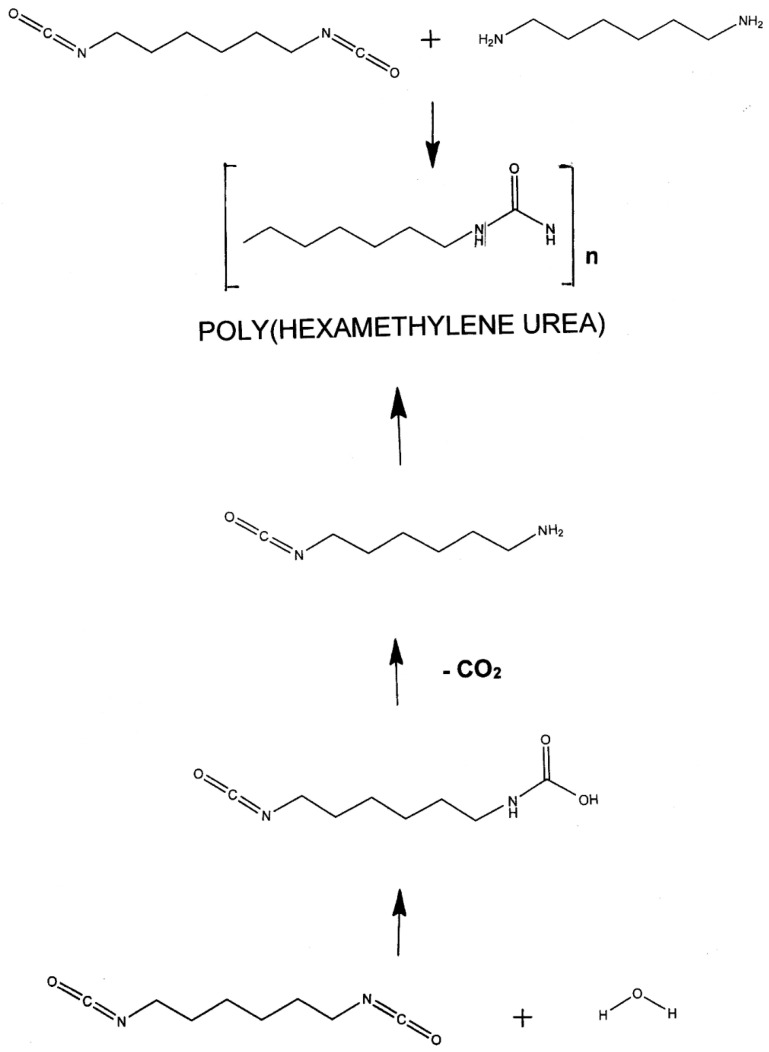
Schematic representation of the synthesis process and theoretical linear chemical structure of poly(hexamethylene urea), as synthesized via the diamine–diisocyanate pathway (upper part—arrow downward) and via the H_2_O–diisocyanate pathway (lower part—arrows upward).

As the exact same polymer can be synthesized via both the water–diisocyanate pathway and the diamine–diisocyanate pathway, these two syntheses pathways and the theoretically linear chemical structure of the resulting poly(hexamethylene urea) are schematically presented in Scheme 1.

The aim of the present research was to investigate and analytically determine the type and extent of occurring side reactions and the resulting chemical structures obtained via the water–diisocyanate polyurea synthesis pathway and to compare the results of the present research to the results of a previously reported analytical investigation of the diamine–diisocyanate pathway synthesis of the same poly(hexamethylene urea) polymer [23].

**Figure 1:**
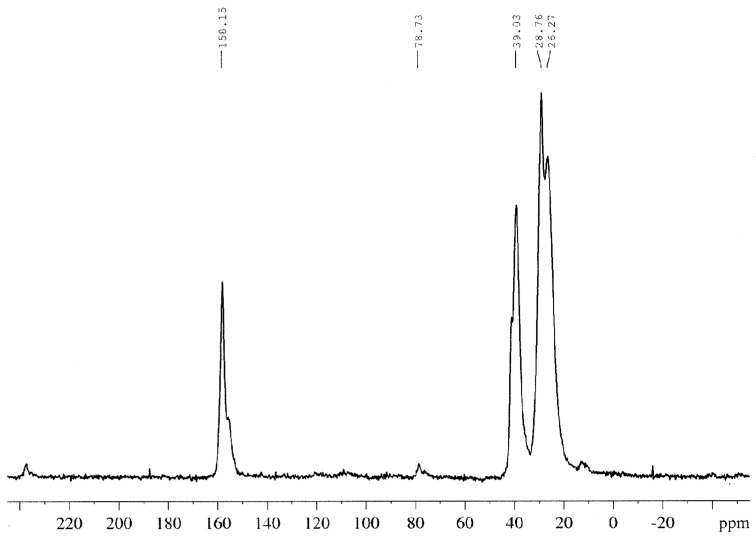
Solid-state ^13^C NMR spectrum of the poly(hexamethylene urea), synthesized via the water−diisocyanate pathway, with H_2_O and HDI at a 1:1 molar ratio.

Figure 1 exhibits the solid-state ^13^C-NMR spectrum of the poly(hexamethylene urea) polymer, hereby synthesized via the water–diisocyanate pathway at a 1:1 HDI: H_2_O molar ratio.

Two types of carbonyl carbon resonances may clearly be recognized in the spectrum. The resonance exhibiting the highest intensity appears at 158 ppm, having a much lower-intensity shoulder to the right at 155 ppm. Nevertheless, according to the theoretically linear chemical structure of this polymer presented in Scheme 1, clearly, only one type of carbonyl should be theoretically present in the polymer. Given the fact that the ^13^C-NMR spectrum exhibits the presence of two types of carbonyls in the polymer—and since in the synthesized polyurea in this study, each carbonyl is necessarily situated between two adjacent nitrogen atoms—the only parameter that may determine the type of carbonyl carbon ^13^C-NMR resonance is whether the adjacent nitrogen atoms are tertiary nitrogens (i.e., tertiary amines) as a result of a further side reaction of the polymer’s secondary amines with isocyanate, or secondary nitrogens (i.e., secondary amines), which have not further side-reacted with isocyanate. 

The side reaction of the secondary amines with isocyanate inevitably involves replacing the H atom on the secondary nitrogen with a carbonyl. Accordingly, the electron density of the carbonyl adjacent to the side-reacted tertiary nitrogens is decreased through the addition of a neighboring electron-withdrawing group. Thus, the carbonyl carbons adjacent to the side-reacted tertiary nitrogens resonate at a higher ppm (i.e., at 158 ppm) than the carbonyl carbons adjacent to the secondary nitrogens (i.e., at 155 ppm). The fact that the intensity of the carbonyl carbons resonance at 158 ppm is very much higher than the intensity of their resonance at 155 ppm indicates that the vast majority of the secondary amines in the polymer had further side-reacted, i.e., the vast majority of nitrogens in the polymer are tertiary nitrogens (i.e., tertiary amines). 

All the additional resonances in the spectrum belong to the six methylene groups of the polymer’s hexamethylene R groups as follows: The resonances exhibiting the strongest intensity are the two very close resonances at around 26 ppm and 28 ppm, belonging to the two inner-most methylenes and to the next two adjacent methylenes of the hexamethylene group, respectively. The resonance at around 39 ppm belongs to the two outer methylenes of the hexamethylene groups, covalently bonded to the nitrogen atoms in the polymer. This resonance exhibits a lower-intensity shoulder resonance at around 41 ppm, which again indicates that these outer methylenes are bonded to either of the two types of nitrogen atoms, i.e., tertiary nitrogens or secondary nitrogens, which is in strong agreement with the above interpretation related to the two types of carbonyl resonances. 

**Scheme 2:**
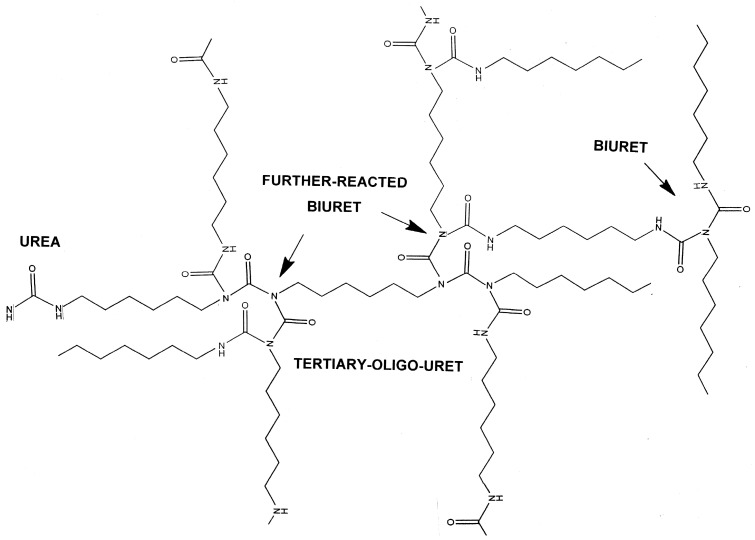
Schematic representation of poly(hexamethylene urea), containing urea, biuret, and tertiary oligo-uret chemical structures (each NH group in the scheme may further react with an isocyanate group, creating a tertiary N and a new NH group that can, in turn, react with an additional isocyanate group, etc., further resulting in hierarchical tertiary oligo-uret structures).

Scheme 2 illustrates a schematic representation of the possible chemical structures that may form as a consequence of the side reactions likely occurring during the polyurea synthesis. 

As presented in Scheme 2, the originally formed urea group exhibits a symmetrical chemical structure consisting of one carbonyl group centered between two N-H groups (i.e., two secondary amines). A nucleophilic side reaction of either one of the urea N-H groups with an isocyanate group results in a biuret structure [1,2,10,22,23]. As exhibited in Scheme 2, this biuret-forming side reaction creates a tertiary amine and a new secondary amine. This newly formed secondary amine can further undergo a side reaction with an isocyanate group, creating an additional tertiary amine and again a new secondary amine, which can undergo a further side reaction with an isocyanate group, etc., consecutively, thus resulting in a tertiary oligo-uret structure [23,24,25]. This is schematically illustrated in Scheme 2. 

The predominance of the occurrence of side reactions and, consequently, the predominance of tertiary amine content in the polymer are strongly reflected in the above-described ^13^C-NMR spectrum (Figure 1). 

**Figure 2:**
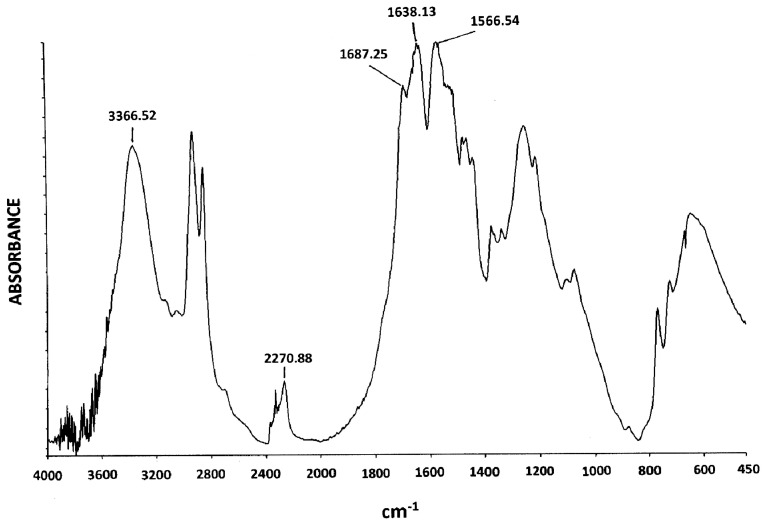
FTIR spectrum of the poly(hexamethylene urea), synthesized via the H_2_O−diisocyanate pathway, with H_2_O and HDI at a 1:1 molar ratio.

Figure 2 exhibits the FTIR spectrum of the poly(hexamethylene urea) polymer, hereby synthesized via the water–diisocyanate pathway at a 1:1 HDI: H_2_O molar ratio.

Two very strong and sharp carbonyl stretching absorbances can be clearly viewed in the spectrum, at 1637 cm^−1^ and 1687 cm^−1^. These two carbonyl stretching absorbances are of almost equal very strong intensity (which indicates their very high abundance in the polymer) (Figure 2). According to the above-described solid-state ^13^C-NMR spectrum (Figure 1), the vast majority of the carbonyls of the polymer are bound to tertiary nitrogens, thus, neither of the two observed carbonyl stretching absorbances in the FTIR spectrum can be due to carbonyls bonded to secondary nitrogens since they are both of almost equal intensity. The absorbance peak of the carbonyls bonded to the secondary nitrogens in the polymer should thus be of much lower intensity (as observed in the solid-state ^13^C-NMR spectrum in Figure 1).

For diisocyanate-derived polyurethanes [24,25] and polyureas [23], it was already previously demonstrated that the carbonyl stretching FTIR absorbance at 1687 cm^−1^ is due to the carbonyls in tertiary oligo-uret structures. Also, regarding polyureas synthesized via the diamine–diisocyanate pathway [23], it was demonstrated that the carbonyl stretching FTIR absorbance at 1637 cm^−1^ is due to the carbonyls in biuret structures. 

Since by replacing the H atom on the secondary nitrogen with a carbonyl, the electron density of the carbonyl adjacent to the side-reacted tertiary nitrogens is decreased through the addition of a neighboring electron-withdrawing group, as explained above, and since several such consecutive adjacent replacements are present in tertiary oligo-uret structures (Scheme 2), the electron density of the adjacent carbonyls is further decreased [23,24,25]. This is in strong agreement with the above-observed location of the tertiary oligo-uret carbonyl stretching absorbance, which is situated at a higher wavenumber (at 1687 cm^−1^) than that of the biuret carbonyls (at 1637 cm^−1^). 

Nevertheless, as indicated by the solid-state ^13^C-NMR spectrum (Figure 1), urea carbonyls should be present in the polymer, albeit in a relatively small amount. In view of the symmetrical urea structure, consisting of one carbonyl group centered between two N-H groups (i.e., two secondary nitrogens), the FTIR urea carbonyl stretching absorbance should appear at a wavenumber that is lower than both the above-described carbonyl absorbances, i.e., as a lower shoulder to the right of the 1637 cm^−1^ absorbance. 

For polyureas synthesized via the diamine–diisocyanate pathway [23], by very rapid early-stage sampling and ice-quenching (and thus decreasing the occurrence of side reactions), it has already been established that the FTIR urea carbonyl stretching absorbance occurs at 1621 cm^−1^ [23]. 

A relatively small absorbance shoulder at this location, however, cannot be observed in the above FTIR spectrum (Figure 2), due to the strong partial overlap of the 1637 cm^−1^ absorbance with the adjacent CNH deformation absorbance at 1566 cm^−1^. 

A new approach for determining the carbonyl stretching absorbance location of the urea groups in the FTIR spectrum of the above poly(hexamethylene urea) polymer is proposed in the present research. This approach stems from a preliminary hypothesis in this study that the very thin surface molecular layer of the polymer that formed in direct contact with the reactor glass may have reacted under different conditions than that of the bulk polymer, due to the direct contact with the highly polar groups of the silicate glass. It was a preliminary hypothesis of this study that the very intimate contact of these surface molecules with the highly polar groups of the silicate reactor glass may have hindered the reactive secondary nitrogens and potentially inhibited the occurrence of further side reactions, thus predominantly yielding urea groups. 

Consequently, in order to analyze this extremely thin outer molecular layer of the polymer, the glass reactor was broken after synthesis completion, and the glass fragments were carefully removed from the polymer surface. An extremely thin nanoscale surface layer of the polymer was tangentially scraped off with a surgical scalpel blade, and a small amount of polymer powder (~8 mg) was obtained and ground with KBr for FTIR analysis. 

**Figure 3:**
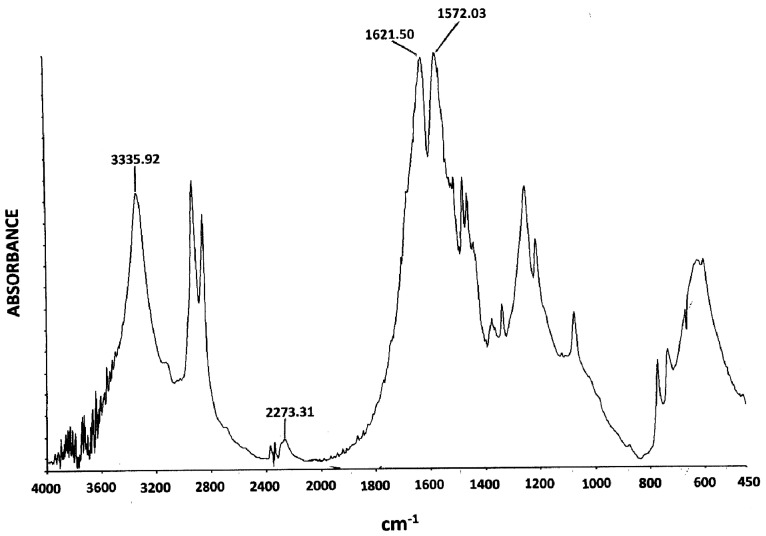
The FTIR spectrum of the very thin outer surface layer of poly(hexamethylene urea), formed in direct contact with the silicate glass reactor wall during the synthesis.

The FTIR spectrum of the resulting material is exhibited in Figure 3. The most evident aspect in the resulting FTIR spectrum (Figure 3) is the single very strong and sharp carbonyl stretching absorbance at 1621 cm^−1^, which may be attributed to the predominantly formed urea groups’ carbonyls and, as predicted above, is located at a lower wavenumber than the absorbances of both the tertiary oligo-uret structure and biuret carbonyl (at 1687 cm^−1^ and 1637 cm^−1^, respectively). This is in strong agreement with the identical location of urea groups’ carbonyl stretching FTIR absorbance, recently demonstrated by performing a rapid very early-stage sampling during polyurea synthesis via the diamine–diisocyanate pathway [23]. 

This result also strongly supports the above-presented initial hypothesis indicating that the very intimate contact of the surface molecules with the highly polar groups of the silicate reactor glass may inhibit the occurrence of further side reactions and therefore predominantly yield urea groups at the synthesized polymer surface. 

The relatively wide base of the 1621 cm^−1^ absorbance peak may indicate the possible additional presence of small amounts of additional types of carbonyls due to the occurrence of a small percentage of further side reactions in these molecules; thus, some degree of crosslinking is still reasonable to occur even here, although shoulders are not observed. 

Additional main absorbances in the spectrum include the very small isocyanate absorbance at 2270 cm^−1^, indicating that most of the HDI molecules in the system reacted; the sharp NH stretching absorbance at 1335 cm^−1^ of the urea groups’ secondary amines; the strong HNC deformation absorbance at 1572 cm^−1^ of the urea groups; the sharp C-N stretching absorbance at 1252 cm^−1^; and the characteristic methylene absorbances belonging to the hexamethylene R groups, i.e., the CH symmetric and antisymmetric stretching absorbances at 2856 cm^−1^ and 2933 cm^−1^, the CH bending absorbance at 1450 cm^−1^, and the CH waging absorbance at around 620 cm^−1^. 

These FTIR results strongly indicate that the majority of secondary nitrogens in the polymer side-reacted to form either biuret groups or further side-reacted to form tertiary oligo-uret structures. This is in high correlation with the above-described solid-state ^13^C-NMR results, clearly indicating that the vast majority of carbonyls in the polymer are bonded to tertiary nitrogens. These results and conclusions are also in strong agreement with the previously published results in polyurethanes [24,25] and polyureas [23], demonstrating the highly preferential occurrence of side reactions in these polymers. 

The observed preferential consecutive occurrence of side reactions between isocyanate groups and secondary amines may be explained by the fact that by replacing an H atom in a urea group with a carbonyl, thus forming a biuret, the electron-withdrawing activity is significantly increased toward the newly formed tertiary oligo-uret structure. This, in turn, increases the polarity of the remaining H atom on the adjacent secondary nitrogen of the original urea group as well as the polarity of the H atom on the other extremity of the newly formed biuret structure (Scheme 2). This significantly increases the reactivity of the newly formed secondary amines toward isocyanate groups. This mechanism may explain the consistently preferential occurrence of these consecutive side reactions, eventually leading to the formation of the tertiary oligo-uret structures.

Nevertheless, the distribution of the two predominant side-reaction-derived structure types, i.e., the biuret and tertiary oligo-uret structures, is very different in the polyurea hereby synthesized via the water–diisocyanate pathway than in the exact same polyurea synthesized via the diamine–diisocyanate pathway, which was previously reported [23]. The polyurea synthesized in this study via the water–diisocyanate pathway exhibited almost equally strong biuret- and tertiary oligo-uret-related FTIR carbonyl stretching absorbances (at 1637 cm^−1^ and 1687 cm^−1^, respectively), while the previously reported polyurea synthesized via the diamine–diisocyanate pathway exhibited almost exclusively a biuret-related FTIR carbonyl stretching absorbance at 1637 cm^−1^ and only a very small absorbance shoulder at 1687 cm^−1^. Thus, it may be deduced here that the much slower kinetics and crosslinking rate of the water–diisocyanate synthesis pathway enabled the sufficiently necessary free volume and steric degrees of freedom for the consecutive formation of large hierarchical tertiary oligo-uret structures. In contrast, the extremely quick reaction kinetics of the diamine–diisocyanate synthesis pathway predominantly enabled the formation of biuret structures and mostly hindered the further formation of the tertiary oligo-uret structures. 

Another interesting aspect that may be observed in the FTIR spectrum of the polyurea synthesized via the water–diisocyanate synthesis pathway (Figure 2) is that the NH stretching absorbance (at 3366 cm^−1^) is very wide. This is in strong contrast with the NH stretching absorbance in the FTIR spectrum of the exact same polymer previously synthesized via the diamine–diisocyanate synthesis pathway [23], exhibiting an extremely thin and sharp absorbance peak. 

Although some effects of hydrogen bonding may not be completely excluded, this very dramatic difference in the width of the NH stretching absorbance peak cannot be significantly attributed to hydrogen bonding effects in this case, due to the following main reasons: The maximum distance range for effective hydrogen bonding is only slightly longer than a covalent bond. Since the present polyurea polymers are very significantly crosslinked, and the length of a crosslinking structure is attributed to at least one hexamethylene group (Scheme 2), the inter-chain and inter-group distances are many times greater than this maximum range. Thus, in this case, hydrogen bonding cannot be considered to have a significant effect on the FTIR absorbances of these polymers. Intra-group (i.e., within the same group) hydrogen bonds may be present in some cases. 

The following explanation for this dramatic difference in the NH stretching absorbance peak width obtained via the two synthesis pathways is hypothesized here: 

As previously reported and described [23], polyurea synthesized via the diamine–diisocyanate synthesis pathway almost exclusively results in the formation of biuret structures. As all biuret structures are exactly the same and exhibit a symmetrical structure (Scheme 2), all the NH groups’ stretching vibration absorbance occurs in practically the same location, resulting in a very thin and sharp absorbance peak [23]. In contrast, the polyurea synthesized via the water–diisocyanate synthesis pathway, as described above, results in highly predominant and almost equal contents of biuret and tertiary oligo-uret structures (Figure 2). The tertiary oligo-uret hierarchical network structures in the polymer can be of various lengths. As the tertiary oligo-uret structure length increases, so does the number of tertiary nitrogens and carbonyls situated between the two terminal NH groups, consequently increasing the electron-withdrawing environment and consequently lowering the NH groups’ electron density (Scheme 2). Thus, the NH stretching vibration absorbance is expected to occur at a slightly different location as a function of the various tertiary oligo-uret lengths in the polymer and at yet a different location for the NH groups in the biuret structures. Accordingly, it is hypothesized here that the very wide NH stretching absorbance in the polyurea synthesized via the water–diisocyanate synthesis pathway most probably stems from the many relatively low-intensity absorbances composing this peak, although shoulders are not observed. 

In order to further elaborate this hypothesis, the synthesis of a completely new type of polymer is hereby presented. 

**Scheme 3:**
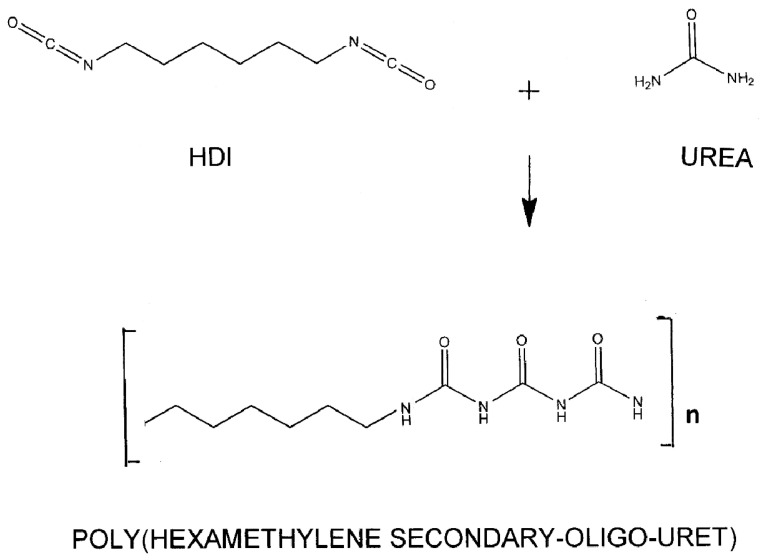
Schematic representation of the synthesis process and theoretical linear chemical structure of the poly(hexamethylene secondary oligo-uret), as synthesized via the diamine–diisocyanate pathway, with urea and HDI at a 1:1 molar ratio.

The synthesis consisted of reacting the same hexamethylene diisocyanate (HDI) with urea at a 1:1 molar ratio. Although urea is actually a diamine—and thus, its synthesis occurs via the diamine–diisocyanate synthesis pathway—the resulting theoretical polymer structure does not form urea groups, but rather secondary oligo-uret structures, which connect the hexamethylene R groups of the polymer. Scheme 3 exhibits the synthesis pathway and the resulting theoretically linear polymer structure.

As observed in Scheme 3, the theoretical linear structure of the resulting polymer inherently exhibits an oligo-uret structure instead of the urea group of the theoretical linear structure of the above-described poly(hexamethylene urea). Nevertheless, since side reactions are inevitably expected to occur, at least part of these consecutively alternating NH groups are expected to further react and result in tertiary oligo-uret structures. 

**Figure 4:**
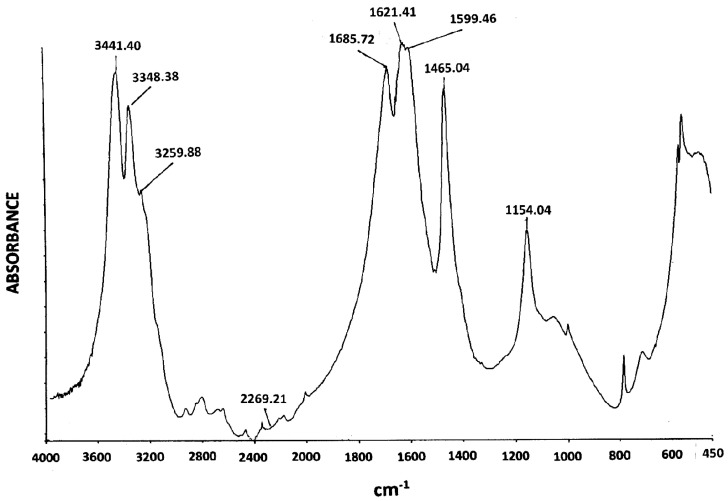
The FTIR spectrum of the poly(hexamethylene secondary oligo-uret), synthesized via the diamine–diisocyanate pathway, with urea and HDI at a 1:1 molar ratio.

The synthesis resulted in the formation of a solid polymer, the weight of which was close to the stoichiometrically calculated weight. Figure 4 exhibits the FTIR spectrum of the resulting poly(hexamethylene oligo-uret) polymer.

**Scheme 4:**
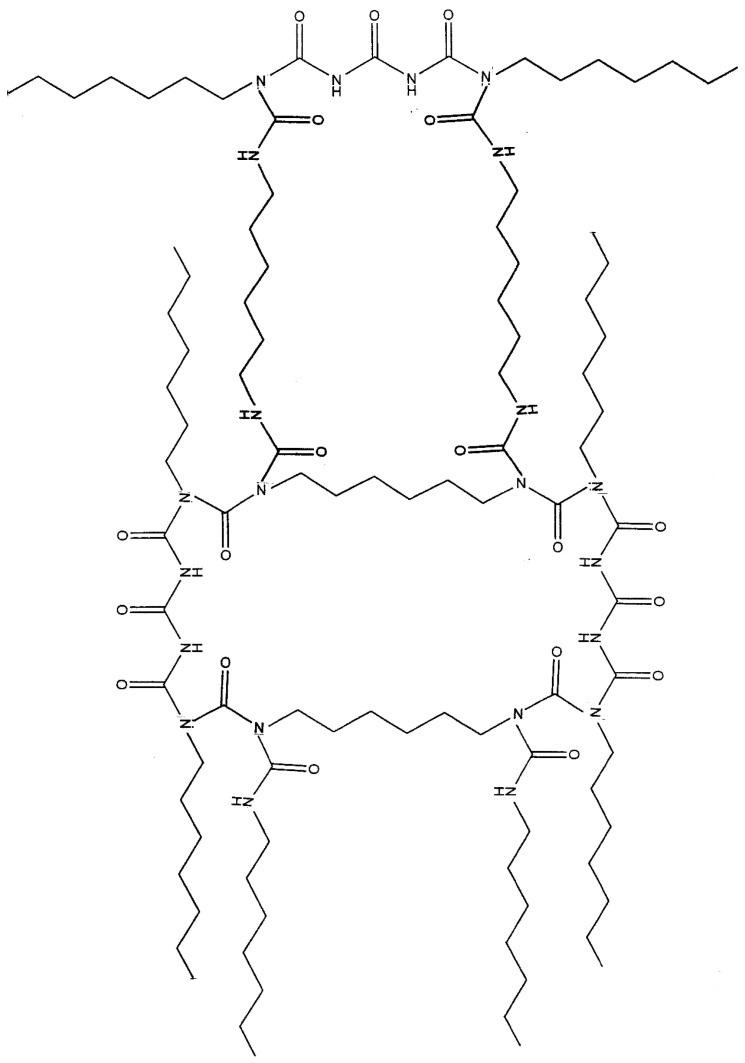
Schematic representation of poly(hexamethylene oligo-uret), exhibiting the product of the side reactions containing tertiary oligo-uret chemical structures and intermittent urea-like structures (each NH group in the scheme may further react with an isocyanate group, creating a tertiary N and a new NH group that can, in turn, react with an additional isocyanate group, etc., further resulting in hierarchical tertiary oligo-uret structures within the sterical proximity hindrance limitations described above).

The very strong and sharp carbonyl stretching absorbance at around 1686 cm^−1^ clearly indicates the predominant presence of tertiary oligo-uret structures in the polymer, indicating the further side reaction of the originally formed NH groups. Nevertheless, the adjacent strong carbonyl stretching absorbance at 1621 cm^−1^ (partially overlapped by the strong CNH deformation absorbance at 1599 cm^−1^), which is characteristic of the urea carbonyl stretching absorbance, as described above, indicates a very significant presence of urea-like structures in the polymer. This diagnostic result is highly consistent with a predominant occurrence of the further side reaction of only two of the four NH groups, which are located at the extreme two sides of the secondary oligo-uret structures (namely the first and fourth NH groups in the structure), most probably due to steric proximity limitations. The occurrence of side reactions at these sites results in the consequent formation of a urea-like group in the center of the structure (as visually presented in Scheme 4), which is highly consistent with and accounts for the strong carbonyl stretching absorbance at 1621 cm^−1^ characteristic of the urea group.

It is interesting to observe the complete absence of the 1637 cm^−1^ absorbance, a characteristic of biuret carbonyl stretching vibrations, which are not present in this new polymer (Scheme 4). 

It is also important to note the presence of three strong and sharp NH stretching absorbances at 3441 cm^−1^, 3348 cm^−1^, and 3260 cm^−1^ with two additional lower-intensity shoulders at 3220 cm^−1^ and 3148 cm^−1^, all partially overlapping and exhibiting a very wide common base. The strong and sharp individual NH stretching absorbances in this spectrum can be accurately observed due to the much higher content of NH groups in the original oligo-uret structures in the theoretically linear repeating unit of this new polymer than in the above-described polyurea. This is in strong agreement with and confirms the above preliminary hypothesis that the very wide NH stretching absorbance in the polyurea synthesized via the water–diisocyanate synthesis pathway most probably stems from the many relatively low-intensity NH stretching absorbances composing this peak, likely resulting from the variations in the tertiary oligo-uret structure lengths along with the quasi-equal presence of the biuret structures. 

Also, in the spectrum, the two very strong and sharp absorbances at 1465 cm^−1^ and 1154 cm^−1^ belong to the symmetric and antisymmetric N-C-N stretching vibrations, respectively. The very small, almost imperceptible isocyanate absorbance at 2270 cm^−1^ in the spectrum indicates that most of the HDI molecules in the system reacted. 

These above-described intermittent urea-like structural intervals between initial side-reaction sites (Scheme 4) most probably provided the necessary free volume that enabled the formation of the complex hierarchical tertiary oligo-uret network structures in this new polymer, despite the extremely quick reaction kinetics and crosslinking rate occurring due to the diamine–diisocyanate synthesis pathway of this polymer. 

**Figure 5:**
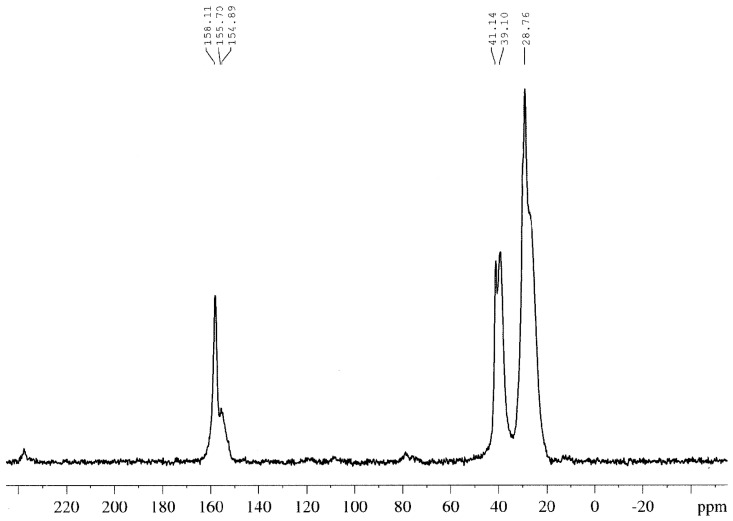
Solid-state ^13^C NMR spectrum of the poly(hexamethylene secondary oligo-uret), synthesized via the diamine–diisocyanate pathway, with urea and HDI at a 1:1 molar ratio.

Figure 5 exhibits the solid-state ^13^C-NMR spectrum of the poly(hexamethylene oligo-uret) polymer, hereby synthesized at a 1:1 HDI–urea molar ratio. 

Two types of carbonyl carbon resonance may clearly be observed in the spectrum: A resonance peak with a higher intensity appears at 158 ppm (which, as described above, can be attributed to the carbonyl carbons bonded to tertiary nitrogens), and a lower-intensity shoulder appears to the right at 155 ppm (which can be attributed to the carbonyl carbons bonded to secondary nitrogens). The fact that the resonance at 158 ppm is of a significantly higher intensity than the resonance at 155 ppm indicates the predominance of further consecutive side reactions occurring following the initial side reactions at two of the four NH groups of the secondary oligo-uret structures (Scheme 4). Nevertheless, it is interesting to note that the intensity difference between the 158 ppm resonance and the 155 ppm resonance in this spectrum (Figure 5) is significantly smaller than the intensity difference between the same resonances in the ^13^C-NMR spectrum of poly(hexamethylene urea) polymer, synthesized via the water–diisocyanate pathway (Figure 1). This may be explained by the more abundant presence of secondary nitrogens in the present new polymer, due to the intermittently formed urea-like structures (Scheme 4), as opposed to the highly predominant tertiary oligo-uret and biuret structures in the poly(hexamethylene urea) polymer, synthesized via the water–diisocyanate synthesis pathway. 

Also, in the solid-state ^13^C-NMR spectrum of this polymer (Figure 5), a resonance peak is observed at 29 ppm, due to the four inner methylene carbons of hexamethylene R groups, and two very close resonance peaks at 39 ppm and 41 ppm are also detected, which belong to the two outer methylenes, connected to the nitrogen atoms (CH_2_N), indicating the presence of the two types of nitrogens in the polymer, namely the secondary nitrogen and the tertiary nitrogen, respectively. 

**Figure 6:**
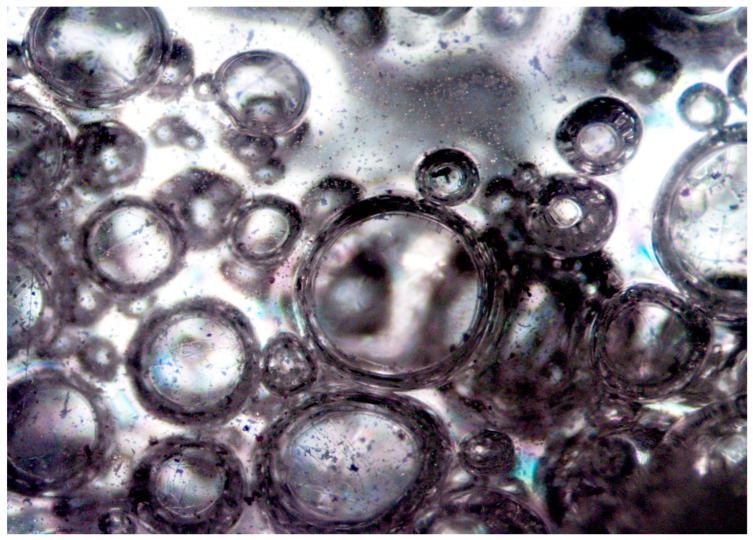
Optical microscopy image of a small thin fragment broken off of the poly(hexamethylene urea), synthesized via the H_2_O–diisocyanate pathway (magnification ×50).

In addition to the resulting chemical structures and crosslinking patterns, another characteristic feature obtained in the polyurea synthesized in this study via the water–diisocyanate synthesis pathway is the foaming (expansion) of the polymer (Figure 6), due to the entrapment of the CO_2_ bubbles released during the first stage of the synthesis. Figure 6 exhibits an optical microscopy image of a small thin fragment broken off of the resulting polymer. 

## 4. Conclusions

Industrial polyureas are typically synthesized using diisocyanates via two possible alternative pathways: the extremely quick and highly exothermal diamine–diisocyanate pathway and the relatively slow and mild water–diisocyanate pathway. 

Although polyurea synthesis via the water–diisocyanate pathway (involving the reaction of diisocyanates and water) is well known and has been industrially applied for many decades, there is surprisingly very little analytical information in the literature in relation to the type and extent of the occurring side reactions and the resulting chemical structures following this synthesis pathway. The present research focused on the direct investigation and diagnostic determination of the occurring side reactions and the resulting chemical structures obtained via the water–diisocyanate polyurea synthesis pathway. 

Both FTIR and solid-state ^13^C NMR analyses indicated a predominant preferential occurrence of side reactions. Solid-state ^13^C NMR analysis indicated that the vast majority of the polyurea carbonyls are bonded to tertiary nitrogens. FTIR spectra indicated a highly predominant, almost equal formation of biuret and tertiary oligo-uret structures, and only a relatively small number of remaining urea structures, which did not further react, the diagnostic FTIR carbonyl stretching absorbance of which was experimentally determined in the present research. 

The results of the present research were compared with those of a previously reported investigation on polyurea synthesized via the diamine–diisocyanate pathway, which, in contrast, exhibited an almost exclusive formation of biuret structures and only a very small number of tertiary oligo-uret structures. It was thus hereby concluded that the sterical degrees of freedom offered by the slower kinetics and crosslinking rate of the water–diisocyanate synthesis pathway enabled the formation of the highly complex hierarchical tertiary oligo-uret structures. 

The suggested mechanisms and the characteristic analytical diagnostic signals’ parameters were further experimentally confirmed and explained through the synthesis and analysis of a completely new type of polymer, inherently exhibiting oligo-uret structures as part of the polymer repeating unit. 

The expanded and highly crosslinked polyurea polymers designed and synthesized in the present research led to the formation of highly rigid, yet very light polymers, which may be suitable for the development of strong engineering structural materials and strong structural biomedical device applications. 

In addition to the enhanced mechanical properties usually associated with polyureas, the polymers developed and synthesized in the present research exhibit significantly enhanced properties due to the fact that these polymers do not contain soft segments and are in fact poly(hard segment) polymers. They also exhibit very significant crosslinking due to the above-described side reactions. The synthesis of these polymers in appropriately shaped molds will provide adequate samples for accurate mechanical assessments and further optimization in specific engineering and biomedical material applications. 

## Data Availability

The data presented in this study are available upon request from the corresponding author.
